# Ozone treatment inactivates common bacteria and fungi associated with selected crop seeds and ornamental bulbs

**DOI:** 10.1016/j.sjbs.2022.103480

**Published:** 2022-11-07

**Authors:** Nedim Çetinkaya, Sercan Pazarlar, İsmail Can Paylan

**Affiliations:** Department of Plant Protection, Faculty of Agriculture, Ege University, Izmir, Turkey

**Keywords:** Ozone, Seed treatment, Bulb treatment, Disinfection, Seed-borne fungi, Seed-borne bacteria

## Abstract

The use of disease-free seeds or bulbs is very crucial to ensure sustainable and profitable agricultural production. Seed-borne pathogens which are responsible for significant yield losses in various crops need to be successfully eliminated with appropriate seed treatments. In this study, we investigated the efficacy of gaseous ozone (O_3_) and ozonated water treatments on the inactivation of seed-borne fungal and bacterial pathogens of widely cultivated vegetable and cereal seeds, and ornamental bulbs. We demonstrated that O_3_ application to tomato and cucumber seeds inactivates *Fusarium oxysporum* f. sp. *lycopersici*, *Fusarium oxysporum* f. sp. *radicis*-*lycopersici*, *Clavibacter michiganensis* subsp. *michiganensis*, *Pseuodomonas syringae* pv. *tomato*, and *Pseudomonas syringae* pv. *lachrymans*, respectively, with no negative effect on seed germination rate. The sterilization capacity of O_3_ has substantially increased when the seeds were soaked in water before the treatments. The saprophytic fungal load and the infection rate of *Pectobacterium carotovorum* subsp. *carotovorum* on several species and cultivars of ornamental bulbs were suppressed by O_3_ treatment. A strong decrease in the infection rate of *Tilletia caries* was also shown in O_3_-treated wheat seeds under field conditions. Overall, the current study indicated that O_3_ treatment has great potential in ensuring the use of disease-free seeds or other propagation materials, which is indispensable at the beginning of crop production.

## Highlights

• Ozone disinfected seed-borne bacteria and fungi from seeds and ornamental bulbs.
• Gaseous ozone showed better performance than ozonated water.
• Pre-soaking the seeds in water increased the efficacy of ozone sterilization.
• Sterilization success of ozone depended on the type of the seed, pathogen, and treatment conditions.

## 1 Introduction

Phytopathogens can attack the reproductive parts of plants during the pre-harvest period in the field, or the products during storage, thereby reducing the quality and quantity of seed harvested (Rennie and Cokerell, 2006, Vega et al., 2019, Gaur et al., 2020, Jagtap et al., 2020). Contamination of seeds or bulbs with some groups of pathogenic microorganisms, such as fungi and bacteria, can limit germination and cause diseases in young seedlings, leading to a significant reduction in plant performance (Chang et al., 2020, Gitaitis and Ronald, 2007). Transmission of seed-borne pathogens during the local and international exchange or trade leads to the introduction of new strains of pathogens that are absent previously, which may subsequently result in the emergence of epidemics (Elmer, 2001, Gergerich et al., 2015, Chalam et al., 2020). Furthermore, some seed-borne fungi such as *Fusarium* sp. and *Alternaria* sp. pose a notable threat to human and livestock health by producing mycotoxins in the seeds (Lee et al., 2015, Oldenburg et al., 2017). Hence, seed treatment is regarded by plant pathologists as an extremely important attempt for eradicating or minimizing seed-borne pathogens from seeds or bulbs.

Chemical-based seed treatment methods, such as the application of disinfectants, fungicides, and bactericides, are frequently used to reduce incidence of pathogens in seeds (Errampalli et al., 2006, Scott et al., 2020, Lamichhane et al., 2020; Molin et al., 2021). In addition to that, alternative physical treatments such as plasma containing energized atoms, UV light, and hot water (thermotherapy), which are considered much more eco-friendly, have also been attempted (Scott et al., 2019, Mangwende et al., 2020, Waskow et al., 2021). Ozone (O_3_) treatment is a fast and economic method to decrease the pathogenic microorganism pressure in drinking and wastewaters, food products such as vegetables, fruits and meat, and food processing equipment (Sopher et al., 2009, Miller et al., 2013, Pandiselvam et al., 2019, Ding et al., 2019, Dong et al., 2018; Khanashyam et al., 2022). O_3_ leaves no residue in the products as it can easily be decomposed into non-toxic products, mainly diatomic oxygen, and has a very short lifetime, making it an alternative green treatment to conventional chemicals generally used in seed disinfection and other agricultural practices (Guo and Wang, 2017, Mohan et al., 2005). The United States Food and Drug Administration approved the ozone for sterilization of water as a safe treatment in 1982 (FDA, 1982). Besides, it is allowed to use in fresh and processed food in different countries (Tiwari and Rice, 2012). The agricultural applications of O_3_ for the management of diseases during the postharvest storage period, decomposing pesticide residues and mycotoxins into nontoxic products, and improving plant growth have attracted attention lately (García-Martín et al., 2018, Chen et al., 2020, Pandiselvam et al., 2020, Liu et al., 2021, Sujayasree et al., 2022). O_3_ can be applied to agricultural products or propagating materials in several ways as gas form or ozonated water, which have advantages and disadvantages depending on the case in process and experimental conditions.

The strong potential of O_3_ in eliminating or reducing food-borne bacteria and fungi in seeds has been demonstrated in the last years. *Escherichia coli*, *Listeria monocytogenes* and *Salmonella* spp. were eliminated from alfalfa seeds with ozonated water treatment without any negative effect on the germinability of the seeds (Sharma et al., 2002, Kwack et al., 2014, Adhikari et al., 2019). Trinetta et al. (2011) showed potential applicability of gaseous O_3_ to reduce *S. enterica* and *E. coli* O157:H7 contamination on pre-inoculated tomato, lettuce, and cantaloupe seeds. Although the seed disinfection potential of O_3_is quite high, there are still few studies on the effectiveness of treatments with O_3_ on seeds harboring plant pathogens. Ciccarese et al. (2007) reported that imbibed wheat, barley, and pea seeds exposed to gaseous O_3_ showed substantial reduction in the population of *Penicillium* spp., *Aspergillus* spp., *Alternaria* spp., and *Rhizoctonia* spp. with no significant effect on seed germination. It has been demonstrated that the treatment of sunflower seeds with gaseous O_3_ reduced the viability of some fungal microorganisms including *Alternaria* sp., *Fusarium* sp., *Aspergillus* sp., and *Penicillium* sp. (Rodrigues et al., 2015). O_3_ has also been shown to be effective in inactivating *Xanthomonas oryzae* pv. *oryzae* (Mohan et al., 2005) and *Fusarium fujikuroi* spores (Kang et al., 2015) in rice seeds. Treatment with O_3_ served as a powerful disinfectant in decreasing the pepper mild mottle virus (Stommel et al., 2021), tomato brown rugose fruit virus (Samarah et al., 2021) and tomato mosaic virus (Paylan et al., 2014) in pepper and tomato seeds, respectively.

Sterilization performance of O_3_ and its effects on seed germination closely depends on the concentration and duration of treatment, the type and structure of the seed, as well as the target pathogen (Mohan et al., 2005, Pascual et al., 2007). Therefore, it is necessary to determine the required concentration of O_3_ and exposure time accurately for each plant species depending on the target pathogen (Pandiselvam et al., 2019). Otherwise, O_3_ treatments can result in unsuccessful sterilization and, more importantly, loss of germination. Here we aimed to investigate the effect of different concentrations of gaseous O_3_ and ozonated water treatments on the inactivation of seed-borne fungal and bacterial pathogens in the seeds of widely cultivated vegetable (tomato, cucumber) and cereal (wheat) and ornamental (tulip, hyacinth, narcissus) bulbs.

## 2 Materials and methods

### 2.1 Pathogens

*Fusarium oxysporum* f. sp. *lycopersici* (*Fol*) isolate BKFol01, *Fusarium oxysporum* f. sp. *radicis*-*lycopersici* (*Forl*) isolate BKForl01 were isolated from tomato seeds and plants collected during field surveys and were stored at 4 °C on potato dextrose agar (PDA) medium. *Clavibacter michiganensis* subsp. *michiganensis* (*Cmm*), *Pseudomonas syringae* pv. *tomato* (*Pst*), *Pseudomonas syringae* pv. *lachrymans* (*Psl*) and *Pectobacterium carotovorum* subsp. *carotovorum* (*Pcc*) isolates were kindly provided by Application and Research Center of Seed Technology, Ege University, Turkey. *Tilletia caries* (*T. caries*) teliospores were collected from seeds of wheat plants growing in the Research and Development Fields of Ege University in 2017.

### 2.2 Source of seeds and bulbs

*Solanum lycopersicum* L. cv. SC2121 (Beta Ziraat©) and *Cucumis sativus* L. cv. Nefes (Bircan Tohum©) were used as vegetable seeds in this study. *Tulipa gesneriana* cv. Claudia, cv. Negrita, and cv. Sogetsu, *Narcissus tazetta* cv. standard, *Hyacinthus orientalis* cv. Aida bulbs were kindly provided by Istanbul Ağaç company (Istanbul, Turkey). *Triticum aestivum* L. cultivars used for field experiments, Cumhuriyet-75 and Gönen-98, were kindly provided by Aegean Agricultural Research Institute, Izmir, Turkey.

### 2.3 Artificial inoculation of seeds

All seeds used in this study were surface sterilized with 70 % ethanol for 1 min followed by a 1 % sodium hypochlorite for 5 min and then rinsed 5 times with sterile distilled water (SDW). After incubation of 24 h either on King’s Medium B (KB) (20 g l^−1^ protease peptone, 1.5 g l^−1^ K_2_HPO_4_, 1.5 g l^−1^ MgSO_4_·7H_2_O, 10 ml l^−1^ glycerol, 15 g l^−1^ agar) or Semiselective *Clavibacter* medium (SCM) (2.62 g l^−1^ KH_2_PO_4_·3H_2_O, 0.5 g l^−1^ K_2_HPO_4_, 0.25 g l^−1^ MgSO_4_·7H_2_O, 1.5 g l^−1^ boric acid, 10 g l^−1^ sucrose, 0.1 g l^−1^ yeast extract, 12 g l^−1^ agar, 100 mg l^−1^ nicotinic acid, 30 mg l^−1^ nalidixic acid, 200 mg l^−1^ cycloheximide), 10 ml of SDW was added on the plates, and cells were dislodged gently by scraping the colony surfaces with a sterile glass rod. The collected bacterial suspensions were diluted to 10^8^, 10^7^, and 10^7^ cfu ml^−1^ for *Cmm*, *Pst*, and *Psl* with SDW, respectively. To obtain microconidia, mycelial plugs (0.9 cm Ø) were transferred from 10 days old fungal culture grown on potato dextrose agar (PDA) to potato dextrose broth (PDB) and incubated 5 days on a rotary shaker at 25 °C and 125 rpm. The conidial suspension was filtered through several layers of cheesecloth, washed many times with SDW and diluted to a concentration of 10^7^ microconidia ml^−1^. Vacuum infiltration was performed as follows; a round membrane filter with a pore size of 0.22 µm (MF-Millipore® Membrane Filter) was placed in a sterile funnel (Sartorius Funnels for Combisart™) mounted on a flask connected to a vacuum pump. The disease-free seeds were placed in the funnel with 50 ml of bacterial or fungal spore suspension and subjected to a pressure of −600 mbar for 10 min at room temperature. The inoculated seeds were air dried overnight under sterile conditions (Ribeiro et al., 2016, Xiulan et al., 2010, Ochi et al., 2017). Control seeds were treated with SDW using the same protocol. Surface sterilized ornamental bulbs were inoculated with *Pcc* by immersing the bulbs in a suspension of bacteria (10^6^ cfu ml^−1^) for 24 h. Inoculation of wheat seeds with teliospore of *T. caries* was carried out by shaking the 1000 g of wheat seeds with 2 g of teliospores in glass bottles for 10 min (Wächter et al., 2008). All test fungi and bacteria were re-isolated from artificially inoculated seeds following the surface sterilization steps and identified via microscopic examination or biochemical tests to confirm that the inoculation was successful, and the pathogens reached the internal parts of the seeds.

### 2.4 Ozone generation and application

Tested concentrations of O_3_ were produced from atmospheric oxygen through a corona discharge type ozone generator (Pap Mobil 2000 Ozone Water Skid, Anseros, Germany) with a constant flow rate of 100 L per hour at 25 °C. Anseros MP 6060 device was used to monitor the concentration of gaseous O_3_ and ozonated water. A PID (Proportional integral derivative) controller was integrated into the O_3_ production system to keep the O_3_ concentrations at desired levels constantly. Artificially inoculated seeds or bulbs were fumigated with gaseous O_3_ at the indicated concentrations in a plastic box with dimensions 39x28x14 cm. Ozonated water treatments were conducted in a tank containing ozonated water (20 °C) by immersing the seeds in permeable bags. Control seeds were either treated with ambient air or tap water without O_3_.

### 2.5 Effects of O_3_ treatment on incidence of pathogens in seeds and bulbs

The elimination success of O_3_ treatments was assessed following the International Seed Testing Association (ISTA) (ISTA, 2010) validated methods. To assess the *Fol* and *Forl* inactivation after the treatment with O_3_, seeds (5 replicates of 10 seeds each plate) were placed on PDA medium and incubated at 25 °C for 5 days and then the fungal growth around the seeds was monitored. Similarly, O_3_ treated seeds were placed on KB or SCM medium and incubated at 28 °C for 48 h to assess the inactivation of *Pst*, *Psl* and *Cmm*. Both fungal and bacterial colonies grown around the seeds were transferred to a new Petri Dish in every experiment and compared with the original culture and confirmed by visual inspection of colonies based on the key morphological characteristics of the microorganism. Seeds with no bacterial or fungal growth were accepted as disinfected ones (Kang et al., 2015). The assessment of the efficacy of O_3_ on fungal load was performed on O_3_ treated seeds and bulbs. Treated seeds and bulbs were brought to the laboratory under sterile conditions. One hundred seeds from each treatment or 10 outer/inner layers of bulbs were immersed in 100 ml of SDW and incubated on an orbital shaker for 30 min at 100 rpm. One ml of properly diluted suspension was spread on the PDA medium and the number of grown colonies after 3 days of incubation at 25 °C were counted and diagnosed. The disinfection capacity of O_3_ against *Pcc* in ornamental bulbs were assessed in storage conditions by counting the number of the decayed bulbs 36 days after treatment.

### 2.6 Evaluation of O_3_ treatment on common bunt disease under field conditions

The efficacy of O_3_ treatments on incidence of common bunt disease was evaluated under field conditions based on the methodology described by Waldow and Jahn (2007). Field experiments were performed at two different locations during 2018–2019 growing season in Izmir, Turkey: Bornova (38°27′05′' N, 27°13′32′' E) and Menemen (38°34′45′' N, 27°01′23′' E). A randomized plot design with four replicates was used at each location. Plots consisted of 1.2 by 3 m with 6 rows and the target field wheat density was 550–600 plants per plot. The number of ears infected with common bunt disease was counted in four rows in each plot and in each row one-meter length was taken. We also included hot water and fungicide treatments in the field studies to compare the efficiency of the O_3_ treatments. Tebuconazole (Raxil DS 2, Bayer Crop Sciences) was applied to teliospore-contaminated wheat seeds at a dose of 150 g/100 kg seed. Seeds were immersed in a hot water at 52 °C for 10 min (Waldow and Jahn, 2007). The yield (kg/plot) and 1000-grain weight (g) were also recorded in the plots remaining after marginal rows were removed.

### 2.7 Effects of treatment on seed germination

The phytotoxicity of the O_3_ treatments on seeds was evaluated with a germination test carried out using the standard “between paper method” described by ISTA (2010). At least 5 replications of 10 seeds were placed between the sterilized and moistened papers and incubated under conditions of 20 °C for 16 h in dark and 30 °C for 8 h in light. Percent of germinated seeds was recorded 10 days after treatment.

### 2.8 Statistical analysis

Data collected from at least two independent experiments were pooled and analyzed using the Graph Pad Prism (v.9). One-way analysis of variance (ANOVA) was used to analyze the influence of O_3_ treatments on seed germination and disinfection, and the mean difference was compared using Tukey’s range *post hoc* test, considering *P* < 0.05 as the threshold for statistical significance. The effects of O_3_ treatments on the saprophyte load of seeds and ornamental bulbs were analyzed with Student’s *t*-test.

## 3 Results

### 3.1 Pre-soaking the seeds in water increased the efficacy of O_3_ in the disinfection of seed-borne pathogens in vegetable seeds

**Fig. 1:**
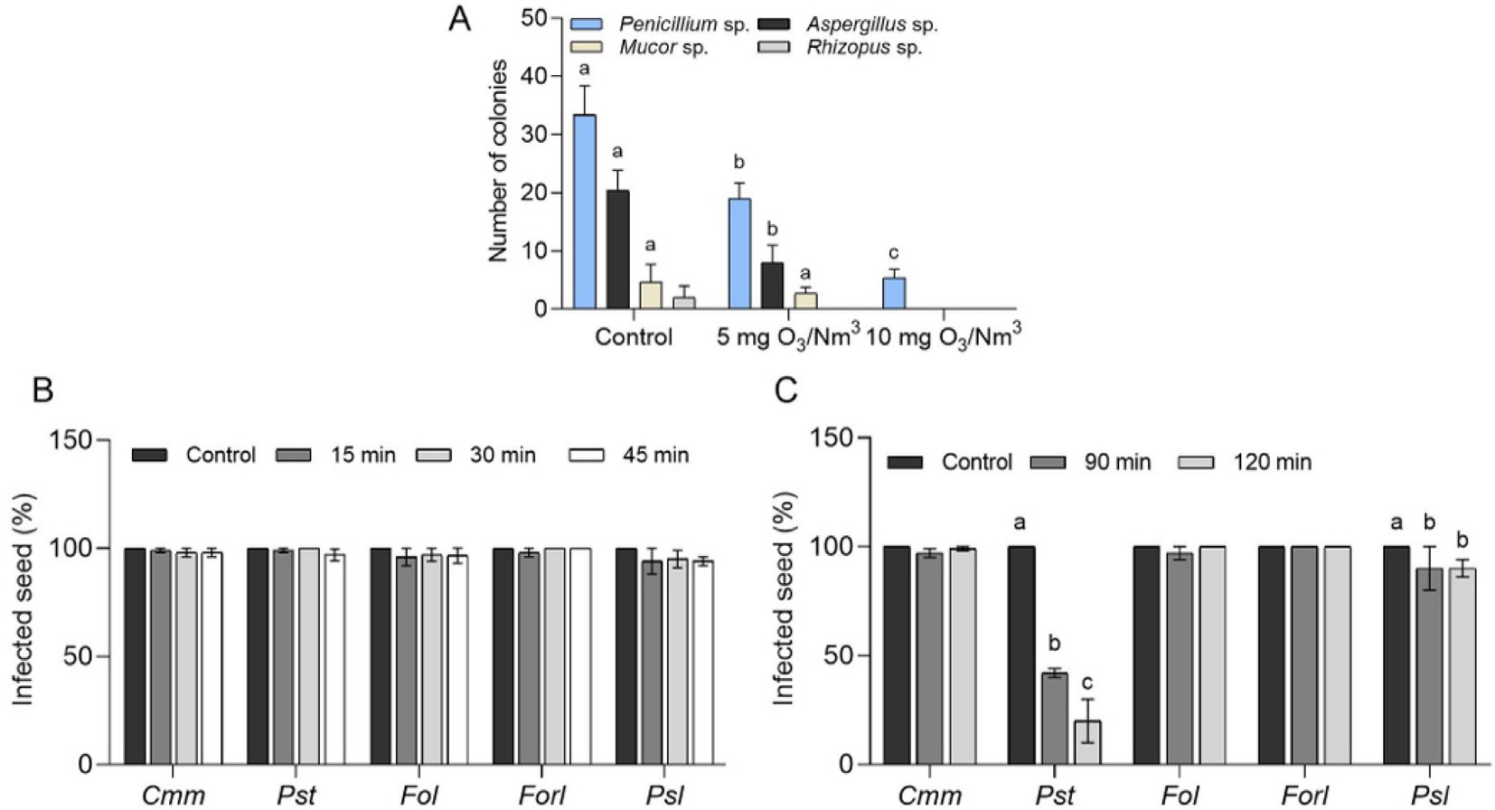
Disinfection efficacy of vegetable seeds by O_3_ treatments. (**A**) Number of the colonies isolated from tomato seeds treated with 5 and 10 mg O_3_/Nm^3^ for 60 min. (**B**) Percentage of infected seeds after treating with ozonated water at a concentration of 8 g O_3_/Nm^3^ for 15, 30, and 45 min. (**C**) Percentage of infected seeds after treating with gaseous O_3_ at a concentration of 150 mg O_3_/Nm^3^ for 90 and 120 min. Different letters indicate statistically significant differences between treatments. Data represent mean ± SE (standard error). *Cmm*; *Clavibacter michiganensis* subsp. *michiganensis*, *Pst*; *Pseudomonas syringae* pv. *tomato*, *Fol*; *Fusarium oxysporum* f. sp. *lycopersici*, *Forl*; *Fusarium oxysporum* f. sp. *radicis*-*lycopersici*, *Psl*; *Pseudomonas syringae* pv. *lachrymans*.

**Fig. 2:**
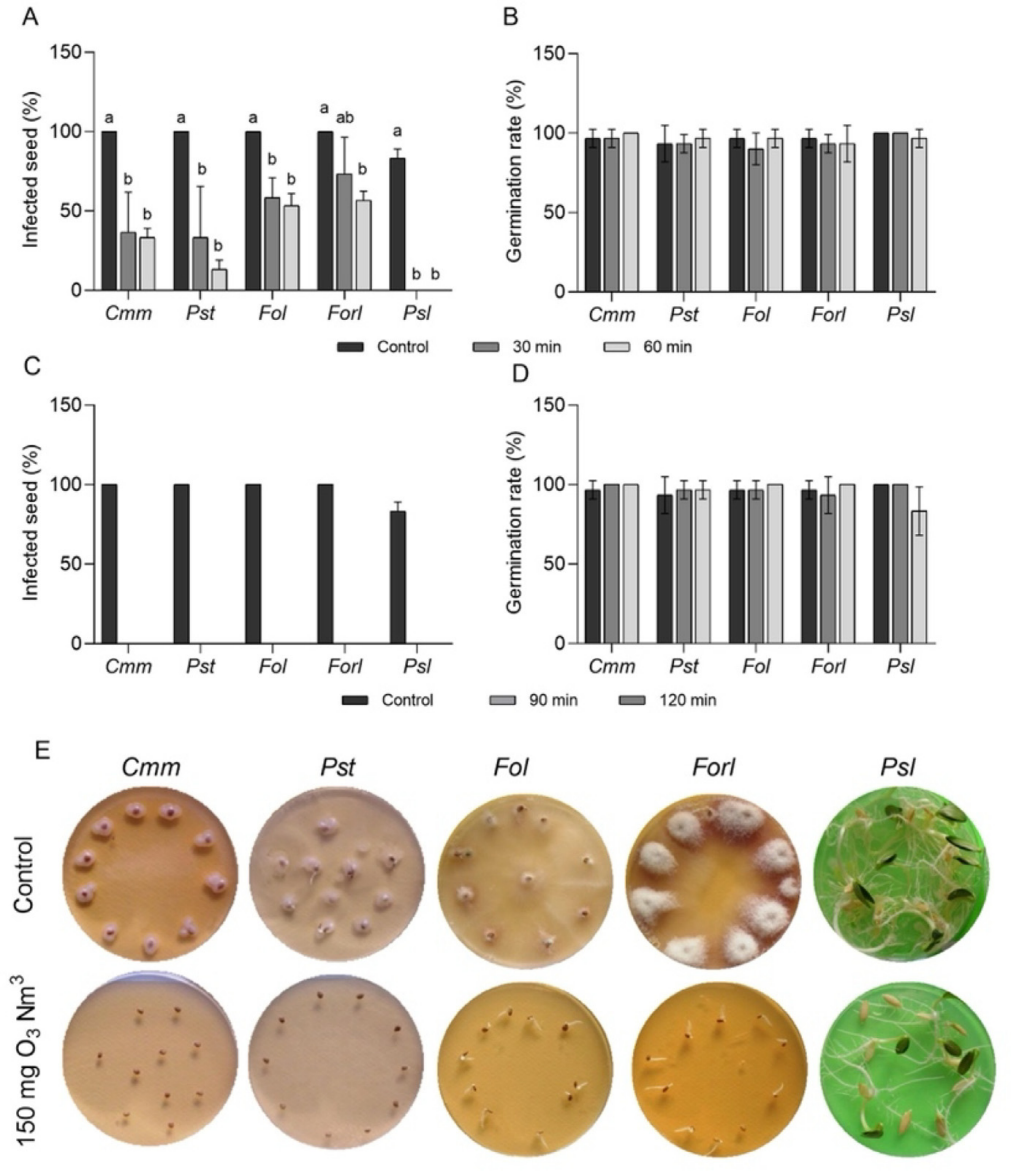
Disinfection efficacy of pre-soaked vegetable seeds in water by O_3_ treatments. (**A**) Percentage of infected seeds after treating with ozonated water at a concentration of 8 g O_3_/Nm^3^ for 30 and 60 min. (**B**) Germination rate of infected seeds after treating with ozonated water at a concentration of 8 g O_3_/Nm^3^ for 30 and 60 min. (**C**) Percentage of infected seeds after treating with gaseous O_3_ at a concentration of 150 mg O_3_/Nm^3^ for 90 and 120 min. (**D**) Germination rate of infected seeds after treating with gaseous O_3_ at a concentration of 150 mg O_3_/Nm^3^ for 90 and 120 min. (**E**) Representative pictures of control and ozone-treated seeds after 5 days post-treatment. Different letters indicate statistically significant differences between treatments. Data represent mean ± SE (standard error). *Cmm*; *Clavibacter michiganensis* subsp. *michiganensis*, *Pst*; *Pseudomonas syringae* pv. *tomato*, *Fol*; *Fusarium oxysporum* f. sp. *lycopersici*, *Forl*; *Fusarium oxysporum* f. sp. *radicis*-*lycopersici*, *Psl*; *Pseudomonas syringae* pv. *lachrymans*.

Gaseous O_3_ application for 1 h at a concentration of 5 or 10 mg O_3_/Nm^3^ was effective in eliminating the saprophyte fungi on seeds which were not surface sterilized (Fig. 1A). There was a significant decrease in the number of *Penicillium* sp., *Aspergillus* sp., *Mucor* sp., and *Rhizopus* sp. colonies on the seed depending on the concentration of O_3_. Seed treatment with ozonated water at a concentration of 8 g O_3_/Nm^3^ for 15, 30, and 45 min did not affect the survival of seed-borne pathogenic fungi and bacteria (Fig. 1B). Gaseous O_3_ treatment at a concentration of 150 mg O_3_/Nm^3^ for 90 and 120 min did not significantly affect *Fol*, *Forl*, and *Cmm* survival; however, it resulted in a slight reduction of *Psl* survival (*∼*10 %) and a striking reduction of *Pst* survival (51.7 % for 90 min and 75 % for 120 min) (Fig. 1C). The germination of seeds was affected neither by ozonated water nor by gaseous O_3_ treatment. Soaking the tomato and cucumber seeds in water for 12 h at room temperature followed by treatment with ozonated water or gaseous O_3_ significantly enhanced the efficacy of disinfection of the seed-borne bacteria and fungi (Fig. 2). Treatment of 8 g O_3_/Nm^3^ ozonated water for 60 min yielded a reduction of 66.7 %, 86.7 %, 46.7 %, 43.4 % and 100 % of *Cmm*, *Pst*, *Fol*, *Forl*, and *Psl* in seeds, respectively, with no negative effect on seed germination rate (Fig. 2A, B). When gaseous O_3_ at a concentration of 150 mg O_3_/Nm^3^ for either 90 min or 120 min was applied to seeds, all seeds were dramatically disinfected from tested seed-borne bacteria and fungi (Fig. 2C, E).

### 3.2 O_3_ exhibited strong potential to control bacterial and fungal pathogens in ornamental bulbs

**Fig. 3:**
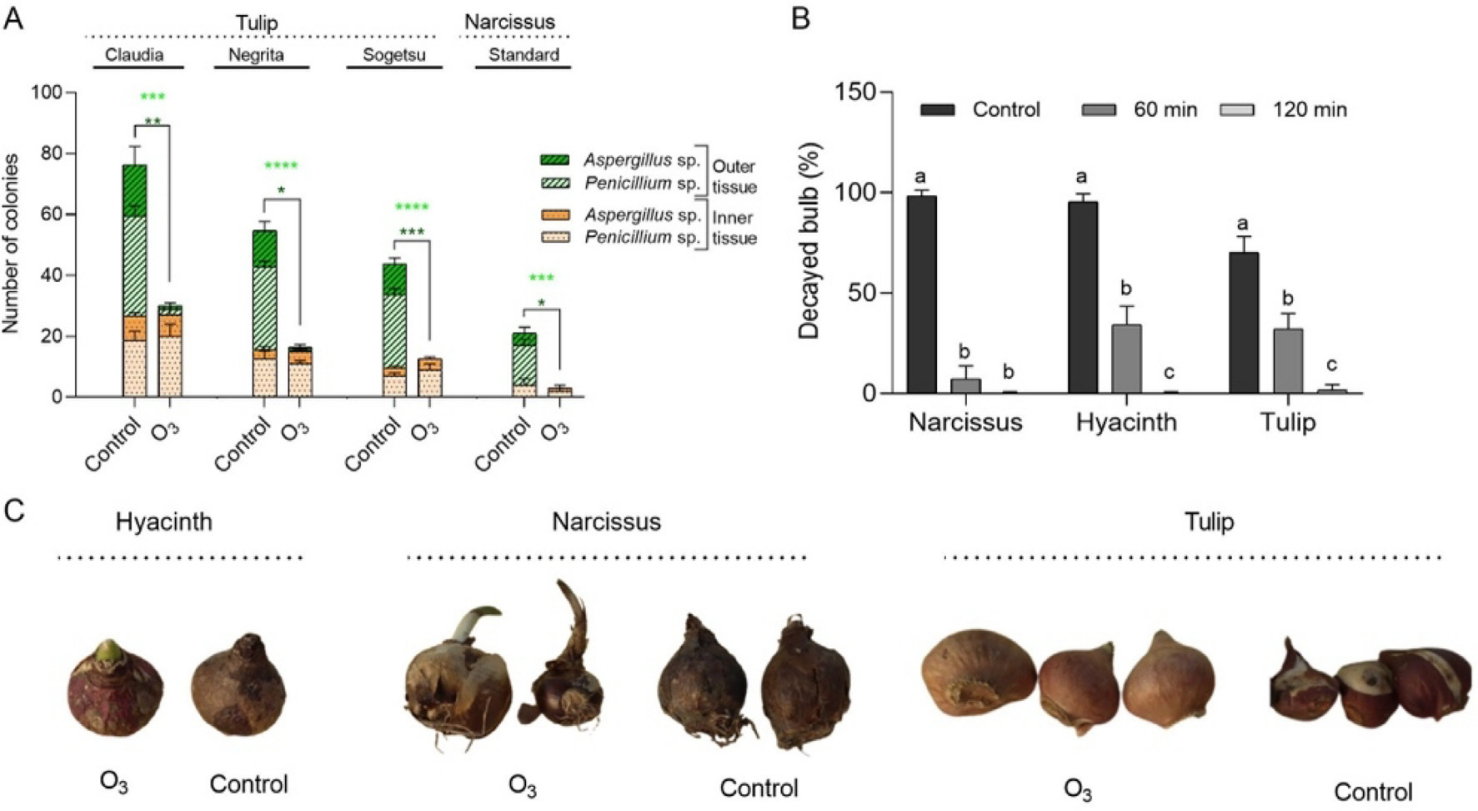
Antimicrobial effects of O_3_ treatment on ornamental bulbs. (**A**) Number of the colonies isolated from outer or inner layers of bulbs treated with 75 mg O_3_/Nm^3^ gaseous O_3_ for 10 min. (**B**) Effects of ozonated water treatment at a concentration of 8 g O_3_/Nm^3^ for 60 and 120 min on the decay rate of *Pcc* inoculated ornamental bulbs. (**C**) Representative pictures of bulbs treated with O_3_ at 36 days post-treatment. Different letters indicate statistically significant differences between treatments. Data represent mean ± SE (standard error). * *P* < 0.05; **P < 0.01, ***P < 0.001, ****P < 0.0001.

Most tulip and narcissus bulbs deposited under unfavorable conditions exhibited abundant *Penicillium* and *Aspergillus* sporulation. Three different tulips and a narcissus cultivars were directly exposed to the 75 mg O_3_/Nm^3^ gaseous O_3_ for 10 min and the number of *Aspergillus* and *Penicillium* isolated from outer and inner layers of bulb tissues was counted. Both *Aspergillus* and *Penicillium* growth on outer tissue of different tulip and narcissus bulbs were inhibited with a success rate of almost 100 % (Fig. 3A). However, the O_3_ treatment at tested concentration was not found effective in eliminating the fungal growth on the inner tissues of bulbs (Fig. 3A). We also tested the potential of ozonated water at a concentration of 8 g O_3_/Nm^3^ for 60 and 120 min for controlling the bulb decay caused by the infection of *Pcc*. O_3_ treatment for 60 min decreased the number of decayed bulbs of narcissus, hyacinth, and tulip by 95.5 %, 64 %, and 53.8 %, respectively. Furthermore, when the treatment duration was increased from 60 min to 120 min, the decrease in the decay rates of narcissus, hyacinth, and tulip increased to 99.6 %, 95.3 %, and 97.6 %, respectively (Fig. 3B, C).

### 3.3 O_3_ treatment to contaminated wheat seeds decreased the common bunt disease in field conditions

**Fig. 4:**
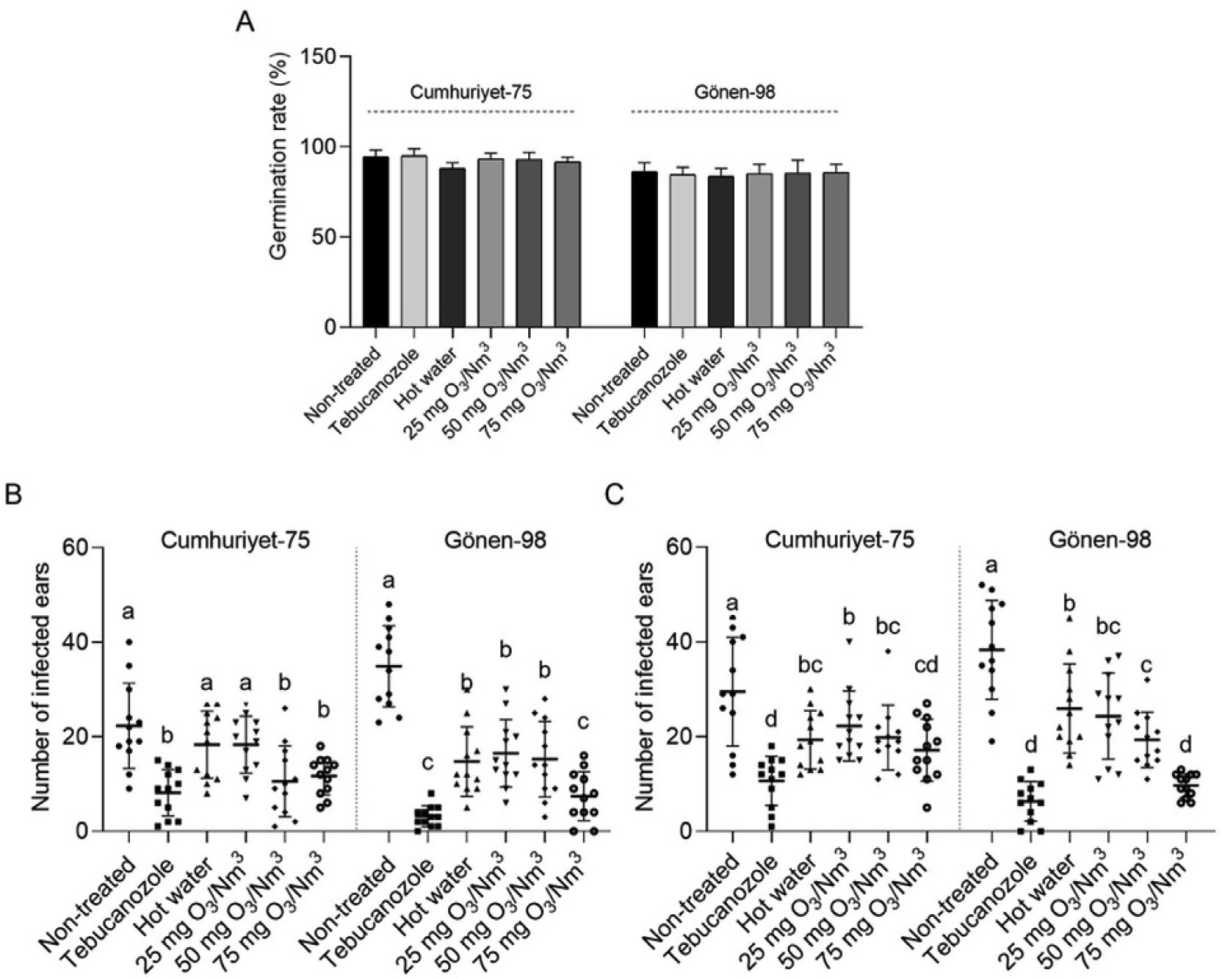
The effects of seed treatment on common bunt incidence in wheat. (**A**) Germination rate of seeds (cv. Cumhuriyet-75 and cv. Gönen-98) after treating with gaseous O_3_ at a concentration of 25, 50, and 75 mg O_3_/Nm^3^ for 60 min, or hot water at 52 °C for 10 min, or Tebuconazole at a dose of 150 g/100 kg seed. (**B**) Number of the *T. caries* infected ears per row at harvest stage in Bornova location. (**C**) Number of the *T. caries* infected ears per row at harvest stage in Menemen location. Different letters indicate statistically significant differences between treatments. Data represent mean ± SE (standard error).

To test whether the gaseous O_3_ as a seed treatment can reduce the common bunt disease, gaseous O_3_ at different concentrations (25, 50, 75 mg O_3_/Nm^3^) was applied to *T. caries* contaminated wheat seeds before planting. No negative effect of the tested O_3_ concentrations on the germination of seeds was found (Fig. 4A). We evaluated the incidence of common bunt disease by counting the number of infected ears in each row at the harvest. There was a significant (*P* < 0.05) difference in the incidence of common bunt disease between most of the treatments as compared to control (Fig. 4B, 4C). The mean value obtained for non-treated control on cv. Cumhuriyet-75 were 22.3 (±2.59) and 29.5 (±3.31) at the Bornova and Menemen locations, respectively. Ozone treatment to Cumhuriyet-75 seeds at a concentration of 25 and 50 mg O_3_/Nm^3^ caused a 17.9 % (18.3 ± 1.75) and 52.6 % (10.58 ± 2.16) reduction of the number of the infected ears in Bornova, and 24.6 % (22.2 ± 2.12) and 32.7 % (19.8 ± 1.97) reduction in Menemen, respectively. Disease incidence in non-treated control plants on cv. Gönen-98 was 34.9 (±2.47) in Bornova and 38.3 (±3.02) in Menemen. 25 and 50 mg O_3_/Nm^3^-treated plants showed less common bunt disease incidence compared to control, by 52.7 % (16.5 ± 2.05) and 56.3 % (15.2 ± 2.30), respectively, in Bornova, and by 36.5 % (24.3 ± 2.61) and 49.7 (19.3 ± 1.68), respectively, in Menemen. The results indicated that seed treatment with 75 mg O_3_/Nm^3^ significantly reduced the number of infected plants in both tested locations and cultivars with a similar success rate of the fungicide treatment. We also evaluated the agronomical traits of wheat plants whose seeds were treated with ozone. Yield and 1000-grain weight increased significantly in both cultivars when the seeds were treated with 75 mg O_3_/Nm^3^ gaseous O_3_ in Bornova and Menemen locations compared to the non-treated control (Table 1).

**Table 1:** Agronomical traits of wheat plants grown in field.

**Cultivar**	**Treatments**	**Bornova**		**Menemen**	
		**Yield** **(kg plot^−1^)**	**1000-grain** **Weight (g)**	**Yield** **(kg plot^−1^)**	**1000-grain** **Weight (g)**
Cumhuriyet-75	Non-treated	0.76 ± 0.05a	37.9 ± 2.4a	0.83 ± 0.08a	35.8 ± 2.2a
	Tebuconazole	1.22 ± 0.05b	52.0 ± 1.0c	1.35 ± 0.05c	50.3 ± 1.2d
	Hot water	0.82 ± 0.08a	43.8 ± 0.8b	0.79 ± 0.10a	42.1 ± 1.1bc
	25 mg O_3_/Nm^3^	0.71 ± 0.07a	44.0 ± 1.9b	0.73 ± 0.08a	40.5 ± 1.1b
	50 mg O_3_/Nm^3^	0.77 ± 0.07a	44.7 ± 0.8b	0.95 ± 0.04ab	43.5 ± 1.6bc
	75 mg O_3_/Nm^3^	0.97 ± 0.13ab	44.0 ± 0.8b	1.13 ± 0.06bc	45.6 ± 1.2c
Gönen-98	Non-treated	0.65 ± 0.12a	24.0 ± 0.7a	0.73 ± 0.03a	25.3 ± 0.6a
	Tebuconazole	1.05 ± 0.04c	34.1 ± 0.8c	1.17 ± 0.03c	30.4 ± 0.8c
	Hot water	0.66 ± 0.08a	24.2 ± 0.7a	0.71 ± 0.05a	25.4 ± 0.5a
	25 mg O_3_/Nm^3^	0.70 ± 0.09a	24.9 ± 0.8ab	0.76 ± 0.08ab	26.3 ± 0.5ab
	50 mg O_3_/Nm^3^	0.59 ± 0.07a	22.7 ± 0.8a	0.85 ± 0.03ab	26.7 ± 1.1ab
	75 mg O_3_/Nm^3^	0.98 ± 0.06b	26.5 ± 0.6b	0.94 ± 0.08b	28.1 ± 1.0bc

Different letters indicate statistically significant differences between treatments.

## 4 Discussion

Disease-free seeds or bulbs are very crucial to ensure sustainable and profitable agricultural production. The limited number of studies conducted so far have shown that O_3_ has potential to eliminate seed-borne pathogens. However, no comprehensive studies show the sterilization capacity of O_3_ forms and concentrations in different pathogens and seeds. In the present study, we investigated the effects of gaseous O_3_ and ozonated water at different concentrations on the elimination of seed-borne bacteria and fungi in vegetable seeds and ornamental bulbs. In addition, we tested the inactivation capacity of seed-transmitted common bunt fungi in wheat by O_3_ treatments under field conditions.

Saprophytic fungi can deteriorate seeds under unfavorable storage conditions, leading to a reduction in seed viability and can cause serious problems in human and animal health by producing mycotoxins (Rajeev and Reddy, 2017, Ramirez et al., 2018). It is also a well-known phenomenon that saprophytic microorganisms in seeds and soil can significantly influence the damage caused by primary pathogens. In this study, we show that gaseous O_3_ even at relatively low concentrations is very successful in reducing the survival of saprophytic fungi, most of which are *Penicillium* sp. and *Aspergillus* sp., on tomato seeds and different ornamental bulbs including tulip and narcissus (Fig. 1A, Fig. 3A). It is not surprising that the very low concentrations of gaseous O_3_ in a short time of treatment is effective to eliminate all the saprophytes which mostly live on the surface of the tomato seeds. However, we observed that saprophyte colonies in inner parts of the bulbs could not be eradicated probably due to the less quantity of the ozone concentrations diffused to inner tissues. A study conducted in barley seeds demonstrated diminished growth of *Aspergillus* sp. when the ozonated water was applied to seeds (Spanoghe et al., 2016). The direct contact of *Penicillium citrinum*, *Aspergillus parasiticus*, and *A. flavus* colonies to gaseous O_3_ was found effective in inhibiting their normal growth (Savi and Scussel, 2014). Both gaseous O_3_ and ozonated water treatment at a concentration of 13.8 mg l^−1^ and 1.7 mg l^−1^, respectively, for 15 and 30 min, completely inhibited saprophytic fungi including *A. flavus*, *A. niger*, *A. parasiticus*, *Byssochlamys fulva*, *Cladosporium cladosporioides*, *Mucor hiemalis*, *M. plumbeus*, and *M. racemosus* in dried figs and both treatments for 180 min resulted in ∼ 90–95 % reduction in aflotoksin B1 level (Zorlugenç et al., 2008). Similarly, Ciccarese et al. (2007) demonstrated that *Aspergillus* spp. and *Penicillium* spp. were successfully eliminated in wheat, barley and pea seeds treated with gaseous O_3_ at a concentration of 3 % by weight for 3 min.

Since the natural infection rate of tomato and cucumber seeds with *Cmm*, *Pst*, *Fol*, and *Psl* was not found high enough to investigate the disinfection capability of O_3_, we used artificially inoculated seeds. Ozonated water treatment did not affect the presence of any seed-borne fungi or bacteria tested, while gaseous O_3_ caused a substantial and slight reduction in *Pst* and *Psl*, respectively, in seeds (Fig. 1B, C). One of the primary findings of this study was that the killing capacity of both ozonated water and gaseous O_3_ is dramatically increased when the seeds were pre-soaked in water. When the seeds were soaked in water for 12 h, ozonated water treatment resulted in different levels of inactivation of fungal and bacterial pathogens depending on microbial species and duration of application, and gaseous O_3_ treatment drastically eliminated all tested seed-borne fungi and bacteria from tomato and cucumber seeds without reducing the seed germination (Fig. 2A-2E). As a technique has been applied for a long while, soaking the seeds in the water for a certain period of time depending on the plant species weaken the seed coats. The increased efficiency of sterilization in water-soaked seeds could be due to enhanced diffusion of O_3_ to internal parts of seeds where the pathogens might be located. The hyphae and preincubated spores are known to be more sensitive to environmental stresses than spores (Levitz et al., 1986, Murdoch et al., 2013). The better performance of O_3_ sterilization in water-soaked seeds than dry seeds may also be due to the cellular activation of fungal spores and bacteria by increased humidity which might result in O_3_ sensitivity. Similarly, a better performance of fungal sterilization by microwave treatment in water-soaked seeds was reported (Mangwende et al., 2020, Szopińska and Dorna, 2021, Cavalcante and Muchovej, 1993).

The mechanism behind the antimicrobial properties of O_3_ is attributed to either its strong oxidizing capacity or reactive oxygen species (ROS) generated during the decomposition (Bocci et al., 2009). Glycoproteins and lipids located in the bacterial and fungal cell membranes are destroyed directly by O_3_ (Cho et al., 2010). Sulfhydryl groups of both apoplastic and intra-cellular enzymes are disrupted by O_3_ and ROS (Menzel, 1971). O_3_ also impairs the integrity of nucleic acids in living cells (Mustafa, 1990). The outermost layer of microorganism cells and their compositions are one of the primary criteria that determine the level of sensitivity to sanitizers including O_3_ (McDonnell and Russell, 2001). The difference in the level of inactivation between pathogens in O_3_-treated seeds could be due to the anatomical or physiological difference between species such as thickness and composition of cell walls or antioxidant capacity of bacteria or fungi involved in scavenging of ROS. In our study, we showed that Gram negative *Pst* presence in non-soaked tomato seeds decreased following the treatment of gaseous O_3_ while Gram positive *Cmm* was not affected (Fig. 1C). This difference might be due to thicker layer of peptidoglycan, which provides physical strength to the bacterial cell wall, in Gram-positive *Cmm* than Gram-negative *Pst*. Several other reports also demonstrated that Gram-negative bacteria are more sensitive O_3_ than Gram-positive ones (Restaino et al., 1995, Moore et al., 2000, Zuma et al., 2009, Rangel et al., 2022). Similarly, the fact that O_3_ in gaseous form did not show any effect against fungal pathogens *Fol* and *Forl* in non-soaked tomato seeds at the tested duration and concentration may be due to the thicker cell wall structure of the fungi. The resistance of fungi or yeast cells to O_3_ has also been shown higher than bacteria cells and viruses (Dyas et al., 1983, Aguayo et al., 2006, de Alencar et al., 2012, Wen et al., 2020). The localization of seed-borne pathogen on seeds, could be either on testa or internal parts of seeds such as endosperm and embryo, closely affect the disinfection success of seed treatments (Koch and Roberts, 2014). However, since we used seeds that were artificially inoculated by using the infiltration technique, the efficacy difference between pathogens is not due to the localization of the pathogens.

One of the other main findings of this study is clearly that gaseous O_3_ is much more effective than ozonated water at tested concentrations in eliminating seed-borne pathogens (Fig. 1, Fig. 2). We tested the maximum concentration of ozonated water as we could reach under our experimental conditions, which is substantially lower than the tested gaseous O_3_ concentration. The high efficacy of gaseous O_3_ than ozonated water was probably due to the difference of tested concentrations. Nevertheless, it should be noted that gaseous O_3_ has almost fourfold greater diffusibility, uniform distribution and penetration ability than ozonated water, resulting in stronger disinfectant ability in most cases (Sapers, 2001, Cullen et al., 2009).

*Pcc* is a well-described Gram-negative bacterium known to cause soft rot in various plants including ornamentals such as hyacinth, tulip, and narcissus. *Pcc* can cause a dramatic loss under favorable conditions if it is not controlled properly (Boyraz et al., 2006, Van Doorn et al., 2008). The promising data found for vegetable seeds motivated us to test whether the ozonated water is effective in disinfecting the bulbs from *Pcc*. Since the bulbs are highly susceptible to gaseous O_3_ (unprovided data), we only tested the ozonated water at a concentration of 8 mg O_3_/Nm^3^ for 60 or 120 min. It was demonstrated that *Pcc* could effectively be disinfected from bulbs by ozonated water treatment (Fig. 3B). The direct toxicological effect of ozonated water against *Pcc* (formerly *Erwinia carotovora* subsp. *carotovora*) and its potential in decreasing leaf symptoms in Chinese cabbage were shown lately (Guo and Wang, 2017).

Common bunt, caused by *T. caries*, is one the important seed-borne disease of wheat in many parts of the world (Gaudet and Puchalski, 1989, McNeil et al., 2004). *T. caries* is mainly managed by synthetic fungicides applied to seeds before planting (Waldow and Jahn, 2007, Matanguihan et al., 2011). Even if the number of infected seeds is very low, seed treatment is highly recommended since the teliospores in infected seeds can easily contaminate the healthy seeds during harvest and storage, and small number of teliospores have strong potential to cause disease. Tebuconazole has been widely used for seed treatment (Hoffmann and Waldher, 1981). In our study, we demonstrated under field conditions that treatment with O_3_ before sowing significantly reduced the number of the common bunt infected spikes and increased the agronomical traits of wheat, with an efficacy rate much better than hot water treatment and almost like systemic fungicide Tebuconazole.

## 5 Conclusion

Synthetic fungicides and disinfectants have been used for seed treatment intensively for several decades to control seed-borne fungi and bacteria. In addition to the negative consequences of synthetic chemicals on human and environmental health, the increasing demand for sustainable and less risky food in recent years has led researchers to seek innovative and sustainable agricultural techniques for efficient production and disease control. Ozone sterilization is an environmentally friendly, effective, relatively cheap, and rapid-acting technique. We showed in this study that O_3_ treatment can potentially replace the fungicide treatments. However, since it is not a selective sanitizer, it carries the risk of damaging non-target biological material such as seeds or bulbs like our case. It should be noted that the reaction of the target seed and the pathogen to O_3_ should be examined very well, and the application conditions should be optimized precisely for successful O_3_ sterilization.

## Funding

This work was supported by the 10.13039/501100004410Scientific and Technological Research Council of Turkey [Grant No 108O380].

## CRediT authorship contribution statement

**Nedim Çetinkaya:** Funding acquisition, Project administration, Supervision, Writing – review & editing, Writing – original draft, Resources, Investigation, Formal analysis, Validation, Conceptualization, Methodology. **Sercan Pazarlar:** Writing – review & editing, Writing – original draft, Investigation, Conceptualization, Methodology. **İsmail Can Paylan:** Funding acquisition, Project administration, Writing – review & editing, Investigation, Methodology.

## Declaration of Competing Interest

The authors declare that they have no known competing financial interests or personal relationships that could have appeared to influence the work reported in this paper.
